# “We don’t want our clothes to smell smoke”: changing malaria control practices and opportunities for integrated community-based management in Baringo, Kenya

**DOI:** 10.1186/s12889-018-5513-7

**Published:** 2018-05-09

**Authors:** Jacinter A. Amadi, Daniel O. Olago, George O. Ong’amo, Silas O. Oriaso, Isaac K. Nyamongo, Benson B. A. Estambale

**Affiliations:** 1 Institute for Climate Change and Adaptation, University of Nairobi, Nairobi, Kenya; 2 Department of Plant Sciences, Kenyatta University, Nairobi, Kenya; 3 School of Biological Sciences, University of Nairobi, Nairobi, Kenya; 4 Cooperative Development, Research and Innovation, The Cooperative University of Kenya, Nairobi, Kenya; 5 Division of Research Innovation and Outreach, Jaramogi Oginga Odinga University of Science and Technology, Bondo, Kenya

**Keywords:** Local knowledge, Malaria trends, Community-based strategies, Framework

## Abstract

**Background:**

The decline in global malaria cases is attributed to intensified utilization of primary vector control interventions and artemisinin-based combination therapies (ACTs). These strategies are inadequate in many rural areas, thus adopting locally appropriate integrated malaria control strategies is imperative in these heterogeneous settings. This study aimed at investigating trends and local knowledge on malaria and to develop a framework for malaria control for communities in Baringo, Kenya.

**Methods:**

Clinical malaria cases obtained from four health facilities in the riverine and lowland zones were used to analyse malaria trends for the 2005–2014 period. A mixed method approach integrating eight focus group discussions, 12 key informant interviews, 300 survey questionnaires and two stakeholders’ consultative forums were used to assess local knowledge on malaria risk and develop a framework for malaria reduction.

**Results:**

Malaria cases increased significantly during the 2005–2014 period (tau = 0.352; *p* < 0.001) in the riverine zone. March, April, May, June and October showed significant increases compared to other months. Misconceptions about the cause and mode of malaria transmission existed. Gender-segregated outdoor occupation such as social drinking, farm activities, herding, and circumcision events increased the risk of mosquito bites. A positive relationship occurred between education level and opinion on exposure to malaria risk after dusk (χ^2^ = 2.70, *p* < 0.05). There was over-reliance on bed nets, yet only 68% (204/300) of respondents owned at least one net. Complementary malaria control measures were under-utilized, with 90% of respondents denying having used either sprays, repellents or burnt cow dung or plant leaves over the last one year before the study was conducted. *Baraza,* radios, and mobile phone messages were identified as effective media for malaria information exchange. Supplementary strategies identified included unblocking canals, clearing *Prosopis* bushes, and use of community volunteers and school clubs to promote social behaviour change.

**Conclusions:**

The knowledge gap on malaria transmission should be addressed to minimize the impacts and enhance uptake of appropriate malaria management mechanisms. Implementing community-based framework can support significant reductions in malaria prevalence by minimizing both indoor and outdoor malaria transmissions.

## Background

Over the past sixteen years, there was a remarkable decline in global malaria transmission [1]. This reduction is attributed to intensified utilization of a combination of primary vector control interventions (Insecticide-Treated Bed Nets (ITNs) and Indoor Residual Spraying (IRS) [2] and accelerated adoption of universal parasitological diagnosis accompanied by admission of artemisinin-based combination therapies (ACTs) [3]. However, the sub-Saharan Africa still leads in global malaria deaths, 92% occurring in 2015 alone [1]. Majority of these deaths occur in rural areas where malaria transmission and burden are high [4, 5]. Malaria is responsible for 18 and 11% of hospital visits in Kenya [6] and Baringo County [7], respectively.

The World Health Organization (WHO) have put in place a multipronged approach for malaria control and elimination, yet in sub-Saharan Africa, the aforementioned malaria control interventions are overemphasized [8]. Malaria prevention and control efforts in Kenya are biased towards lake endemic and highland epidemic zones [9], with least effort in other malaria endemic zones. One of the neglected but important zone in terms of malaria transmission is Baringo County; a county categorized as a seasonal malaria transmission zone [9].

Studies have shown that the use of ITNs, IRS and malaria treatment interventions significantly reduce malaria cases [10, 11]. However, the aforementioned strategies have never been shown to eliminate malaria transmissions [12]. For example, a combination of numerous strategies contributed to malaria elimination in Europe including a combination of vector control through larval source management, disease and vector surveillance and robust financial investment [12]. In the tropics, residual malaria transmission may render malaria elimination unattainable without improved or new vector control strategies [13, 14]. Integrated vector management (IVM) that incorporates complementary vector control methods such as larval source management to the principal strategies remains the most sustainable malaria control strategy [15]. The supplementary vector control options when integrated with principal interventions may substantially reduce residual malaria transmission at local level [16].

Local communities are at the core of any risk management plan. Their perception, views, and understanding of the problem is crucial in designing actionable solutions. In Kenya, malaria control has been a top-down initiative primarily by use of Long Lasting Insecticidal Nets (LLINs), IRS and case management strategies [17]. In the semi-arid zones, the government recommends the use of LLINs among pregnant women and children under one year [9] contrary to the whole population as recommended in malaria endemic zones. Malaria transmission occur both indoors and outdoors in Kenya [18]. A shift from indoor to outdoor malaria transmission that is linked to the changes in vector ecology has been reported [19]. *Anopheles arabiensis* the dominant vector in Baringo [20] predominantly rests and feeds outdoors implying a greater risk of outdoor malaria transmission. Baringo County has malaria prevalence of 2.6–10.5% yet, a recent study found that perennial transmission occur in lower altitudes especially the riverine zone [21]. This suggests that the current interventions may be ineffective in reducing malaria prevalence since outdoor malaria transmission remain largely unabated [22]. In light of challenges such as insecticide resistance, financial constraints, and inadequate LLINs [2, 5], community-driven integrated approaches to malaria control should be explored. This study presents a community-focused approach for malaria reduction by addressing the following objectives: analyse malaria trends; assess lay knowledge on causes and prevention of malaria and develop malaria control framework for communities in Baringo County.

## Methods

### Study area

**Fig. 1:**
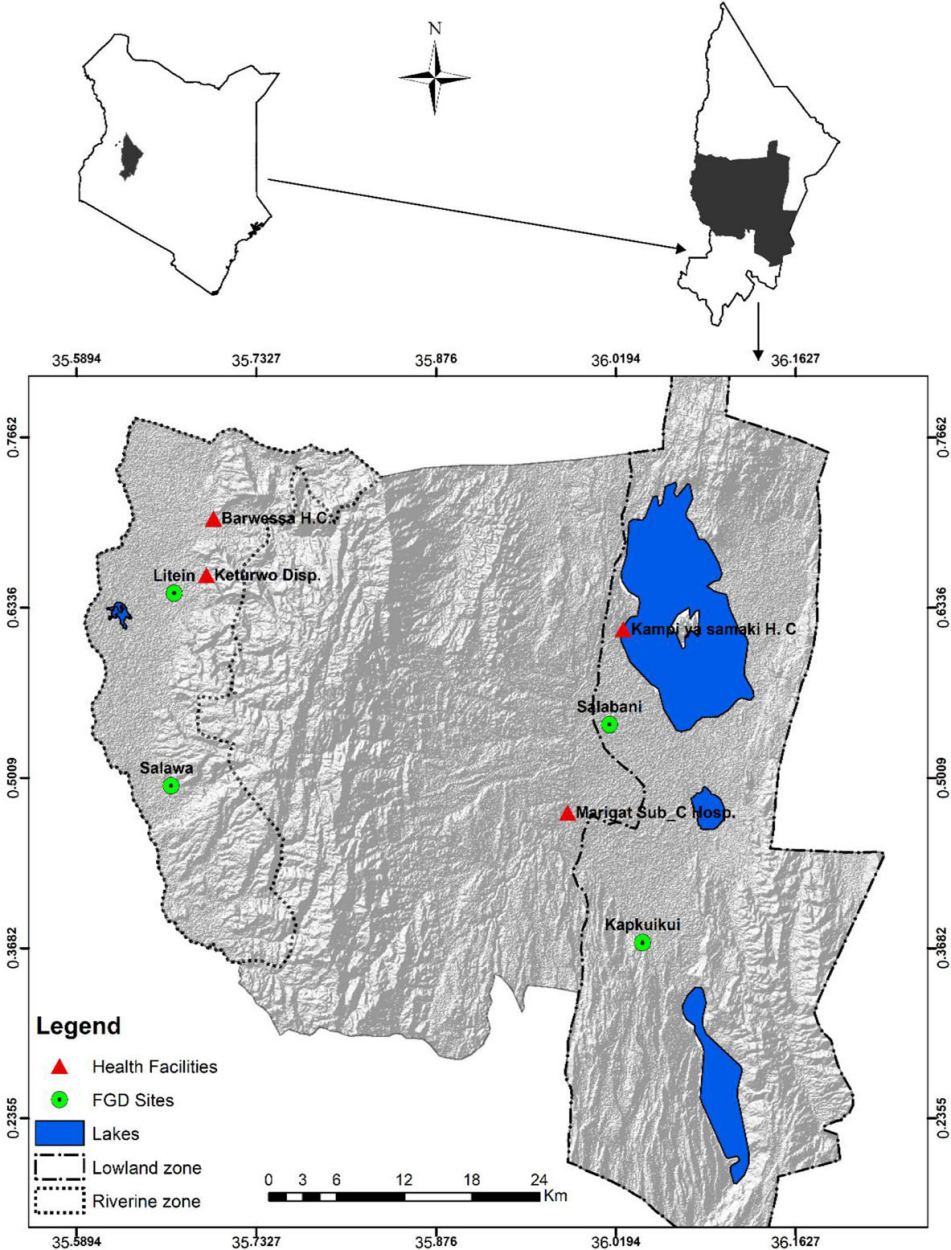
Map of Baringo County (Kenya) showing selected ecological zones, health facilities and focus group discussions (FGD) sites

Baringo County Kenya is classified into four ecological zones based on altitude (900–2300 m a.s.l.), vegetation types and climatic characteristics. The daily air temperature ranges between 16 °C and 42 °C while mean monthly minimum and maximum temperatures are 20 °C and 35 °C respectively. Average annual rainfall ranges between 300 and 600 mm. The county is largely rangeland with pastoralism and irrigation farming being the key economic activities*.* The county has a population of 555,561 and poverty levels estimated at 58.5% as reported during the 2009 Kenya National Census. Tugen and Ilchamus communities dominate the region. In this study, communities in two ecological zones, riverine (1100–1200 m a.s.l.) and lowland zones (below 1000 m a.s.l.; Fig. 1) were selected based on matrix ranking. A matrix ranking tool uses multiple attributes in decision making and prioritization [23].

### Malaria data collection

Records on clinical malaria cases for the period 2005–2014 from four health facilities namely Barwessa and Keturwo dispensaries in the riverine zone, and Kampi ya Samaki health centre and Marigat sub-county hospital in the lowland zone (Fig. 1) were obtained through the Department of Health Services. During data abstraction, unique codes were used to replace names in the records. Distribution of malaria cases per location for the year 2014 was generated using ArcMap 10.0.

### Study and sampling design

Mixed-methods approach is known for its contribution to a holistic understanding of the subject of inquiry. In this study, a combination of FGDs, key informant interviews (KIIs) and household survey questionnaires were used to gather information on local knowledge on causes and prevention of malaria and in developing integrated malaria control framework for communities in Baringo County. First, eight (four gender-specific) FGDs were conducted using a semi-structured interview guide. Villages were divided into strata and 86 people (47 women and 39 men) purposively selected participated in the discussions based on the duration they had lived in the area (more than 10 years) and their personal experiences with climate hazards and malaria. The FGD guide was pretested in one female-only FGD in Barwessa to assess content suitability, participatory processes, duration and to identify emerging issues. The discussion sessions lasted between one hour and one hour-fifteen minutes, during which the data was captured using a combination of audio records and note taking. The outcomes of the FGDs were used to establish important themes covered during interviews. Twelve (12) key informants (Department of Health Services and community resource persons) were purposively selected based on their role in the community. The interviews were conducted during September–October 2016.

Semi-structured questionnaires were administered to 300 randomly selected households (150 per zone); sample size generated using eq. 1 as used by Krejcie and Morgan [24]. The questionnaires were first pretested in Marigat and Barwessa using a total of 30 respondents. The two sites were later excluded from the actual survey. Requisite changes were made on the questionnaire before actual data collection. Data collection was conducted by Tugen and Ilchamus speaking enumerators.

Eq. 1 $$ s={X}^2 NP\left(1-P\right)\div {d}^2\left(N-1\right)+{X}^2P\left(1-P\right) $$

Where:

*s* ~ required sample size,

*X*^*2*^ ~ the table value of Chi square for one degree of freedom at 95% confidence level (3.841).

*N* ~ population size.

*P* ~ the proportion in the target population estimated (assumed to be 0.5 that generates maximum sample size).

*d* ~ the degree of accuracy expressed as a proportion (0.05).

### Stakeholder identification and framework development

Stakeholders were purposively selected and participated in the development of a malaria risk reduction plan. The local county administrators aided the identification of the stakeholders. Two stakeholders’ consultative forums were conducted in Salawa and Marigat in May 2017. Twenty-one (21) and 25 stakeholders (community resource persons, officials from the Department of Health Services, local administration and representatives from community-based organizations) drawn from riverine and lowland zones, respectively participated during the forums. The malaria risk reduction plan for communities in Baringo was developed based on the processes outlined in the Community Based Disaster Risk Management (CMDRM) framework [23], the Kenya Malaria Strategy (KMS) 2009–2018 [25] and Malaria Communication Strategy (MCS) 2016–2021 [26]. A combination of ranking, seasonal calendars and group discussions were used during malaria risk assessment and planning process. The report from discussions was provided to stakeholders for validation and revisions were made based on feedback.

### Data analysis

Missing data on the numbers of malaria cases was 3.5% (17 out of 480 months) and was imputed using the Multivariate Imputation by Chained Equations (MICE) package in R software. Malaria trends for the 2005–2014 period were analyzed using the Mann-Kendall and Seasonal Mann-Kendall tests in R version 3.2.2 software [27]. Household survey data was entered, cleaned, and analyzed in STATA version 13.1. Chi-square test (*p* < 0.05 significance level) was used to test for independence between respondents characteristics and their opinions on malaria transmission and risk factors.

Audio files from FGDs and KIIs were transcribed and each script verified by comparing the notes to the transcribed scripts. This was followed by data cleaning and coding into emergent themes using a content analysis method in N-vivo version 10.

## Results

### Malaria trends during 2005–2014 period

**Fig. 2:**
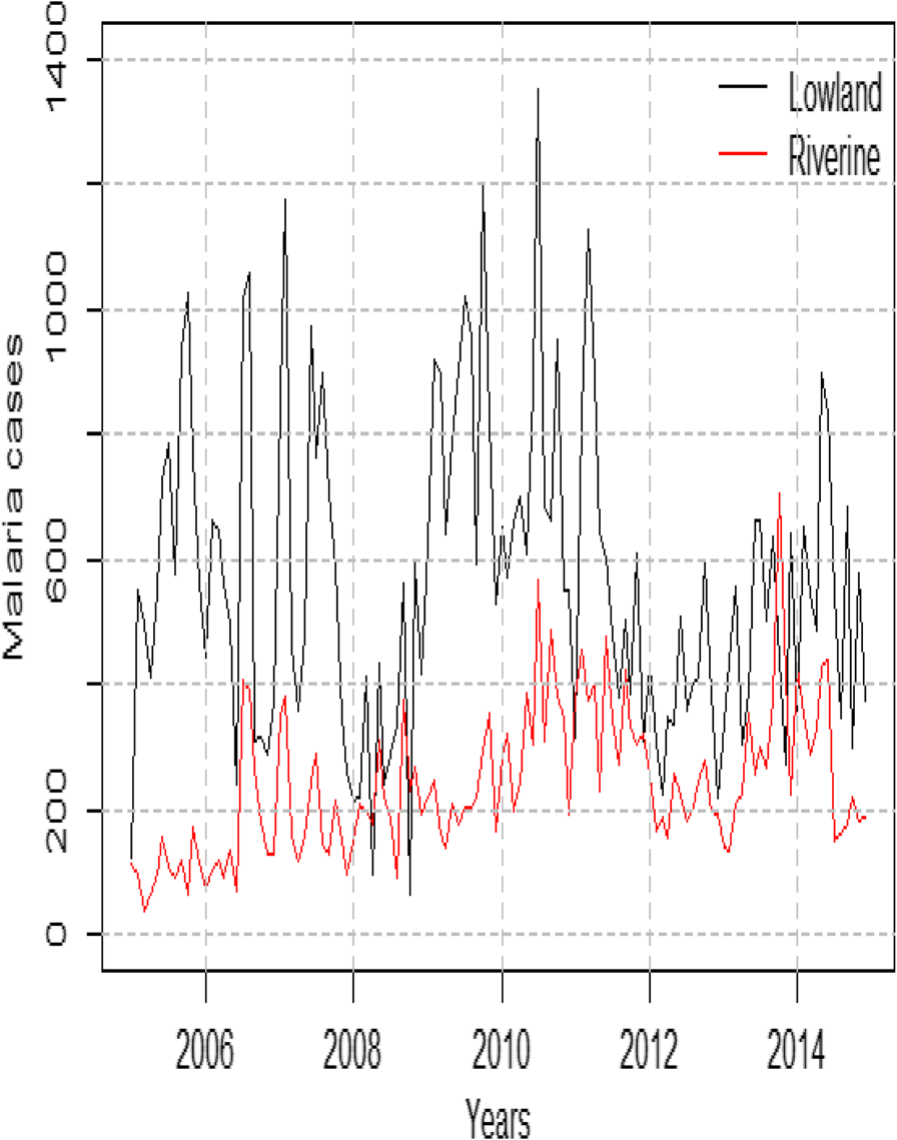
Trends in malaria cases in the riverine and lowland zones during 2005–2014 period

**Fig. 3:**
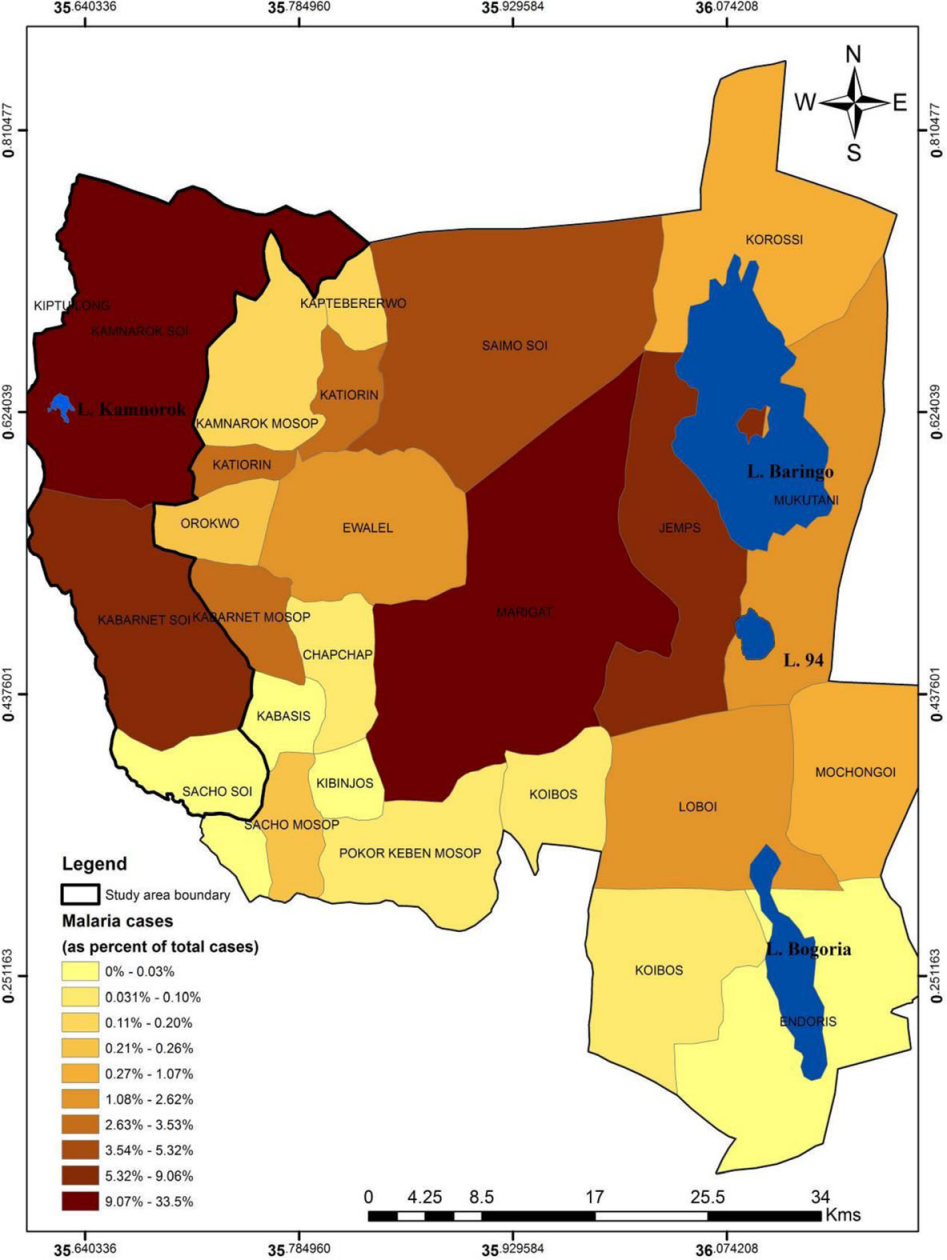
Total annual malaria cases per location for the year 2014

Statistical analysis revealed varied malaria trends in the surveyed zones (Fig. 2). In the riverine zone, malaria cases increased during the 2005–2014 period (tau = 0.352; *p* < 0.001) while a decrease was observed in the lowland zone (tau = − 0.076; *p* > 0.05; Fig. 2). High malaria cases were observed in Kamnarok Soi and Kabarnet Soi locations in the riverine zone as well as in Marigat and Njemps locations in the lowland zone during the year 2014 (Fig. 3). These findings concurred with observations by a key informant as expressed in the following statement:

‘*Actually, malaria trends have been high in some areas in Kerio valley (riverine zone) including Kabutiei, Kaboskei, and Lawan. When you look at it (malaria trends) it is actually continuous. It’s not seasonal within Kapluk, Katibel, L. Kamnarok, Litein, Keturwo, Barwessa, Kiplolwon, Chemoso and Kuikui areas*’. Key informant, sub-county malaria control unit.

A significant increase in seasonal malaria cases was noted in the riverine zone (tau = 0.418; *p* < 0.001), the long rains (March, April and May) season having significant increases compared to other seasons (tau = 0.733; *p* < 0.01). However, there was no change in seasonal malaria cases in the lowland zone. A significant increase in monthly malaria cases was observed during March, April, May, June and October in the riverine zone as opposed to the lowland zone (Table 1).

**Table 1:** Trend in monthly malaria cases during 2005–2014 period

	Lowland zone		Riverine zone	
Months	Tau	*p*	Tau	*p*
January	0.02	1.00	0.42	0.11
February	−0.11	0.72	0.29	0.29
March	0.02	1.00	0.67	0.0092**
April	0.02	1.00	0.69	0.0073**
May	0.02	1.00	0.73	0.0042**
June	0.02	1.00	0.56	0.032*
July	−0.20	0.47	−0.07	0.866
August	−0.38	0.15	0.09	0.79
September	−0.07	0.86	0.20	0.47
October	−0.20	0.47	0.56	0.032*
November	−0.11	0.72	0.33	0.21
December	−0.02	1.00	0.49	0.059**+**

Notes: *p* is the significance level: ‘+’ 0.1, ‘*’ 0.05, ‘**’ 0.01; tau is the Mann Kendall’s statistic for trend analysis. A negative symbol (−) denotes a decreasing while a positive symbol denotes an increasing (+) trend

### Characteristics of respondents

Respondents aged between 20 to 89 years, with a mean age of 44 (Table 2). Fifty three percent (53%; 159/300) had primary school education while 22% had no education. The main livelihood activities of the communities were crop farming and livestock rearing at 54.7 and 18.3% respectively.

**Table 2:** Demographic information of survey respondents (*n* = 300)

Gender	Male		Female
Lowland	66 (44.0%)		84 (56.0%)
Riverine	73 (49.0%)		77 (51.0%)
Total	139 (46.3%)		161 (53.7)
Level of Education	Total frequency (%)	Gender	
		Male (%)	Female (%)
None	65 (21.7)	21 (32.3)	44 (67.7)
Primary	159 (53)	70 (44.0)	89 (56.0)
Secondary	53 (17.7)	31 (58.5)	22 (41.5)
Middle-level College	20 (6.7)	15 (75.0)	5 (25.0)
University	3 (1)	2 (66.7)	1 (33.3)

### Local knowledge on malaria transmission and risk factors

**Fig. 4:**
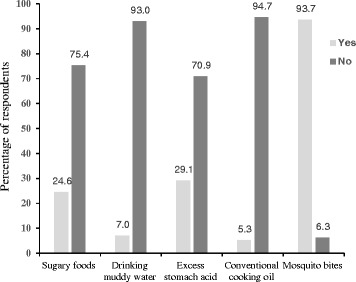
Causes of malaria

Nearly all (93.7%) survey respondents were aware that malaria is transmitted by a mosquito bite (Fig. 4). However, misconceptions on the cause of malaria existed. For example, during FGDs, some people believed that drinking muddy water, excess stomach acid “nyongo”, eating sugary foods, or eating food cooked with conventional cooking oil caused malaria as stated in the quotes:

‘*Then we would drink that (muddy) water and be infected with malaria*.’ Female participant, Salawa.

‘*You clear off the green substance (algae) then fetch the water. It would take you a few hours to fall sick*.’ Female participant, Salawa.

About 95% of survey respondents agree with the opinion that staying outdoors after dark exposed someone to mosquito bites. Gender or age did not influence opinions on outdoors malaria transmission. However, the level of education was positively associated with the opinion that staying outdoor increased the risk of mosquito bite. People with higher education were more confident that staying outdoors was a factor that exposed someone to malaria risk (χ^2^ = 2.70, df = 4, *p* < 0.05).

Various chores and social activities were identified to increase the risk of out-door malaria transmission. For example, during in-depth interviews, older men were reported to engage in politics and social drinking till late in the night. In FGDs, it was reported that open air traders stayed outdoor up to 2200 h selling commodities. Informants identified irrigated crop farming as a possible risk factor for malaria transmission among people living in the lowland zone. In Perkerra, Njemps, Eldume, and Loboi, men preferred watering their crops at night prolonging their exposure to mosquito bites. In the riverine zone, older boys and men stayed overnight in the farms protecting crops against wild animals such as elephants. This was common during the harvesting period in August and September when crop depredation caused immense loss and would be prevented at all cost. This is as expressed in the following statement:

‘*They (men) are forced to stay in the farms to protect their crops such as maize because of these wild animals. So, people stay overnight in the farms even for four weeks, sleeping on trees*.’ Community resource person, riverine zone.

Other activities such as going to the farms early in the morning (0600 h), circumcision ceremonies and livestock herding were mentioned by informants as predisposing factors to malaria transmission. During circumcision ceremonies, boys aged between 13 and 18 years together with older men spent about four weeks in the wilderness. It was reported that during these ceremonies, participants did not use any form of malaria prevention or treatment. In the lowland zone, warm weather condition was additional reason why people stayed longer outdoors during early evening. Furthermore, women and girls conducted most of their cooking while outdoors.

Results show that about 59.6% (179/300) of survey respondents did not protect themselves while outdoors. There was a gender differentiation among those who protected themselves, 67% being women while 33% were men. About 40.4% of those who protected themselves while outdoors at night used additional clothing like socks, long pants and long-sleeved clothes.

### Existing strategies for malaria control

#### Conventional methods

About 68% (204/300) of survey respondents owned a mosquito net majority being women. It was reported that mass net distributions were conducted in areas perceived as malaria hotspots in the lowland zone contrary to the riverine zone. The biased mass net distribution was revealed by a key informant as illustrated:

*‘Recently, I was arguing in a certain meeting why Marigat had mass net distribution yet there are areas such as Salawa, Kuikui, Kapluk, and Barwessa (in the riverine zone) that have malaria episodes almost year-round. But I was told the data from Kerio Valley does not support what I was saying.*’ Key informant, County Disease Surveillance.

This study revealed that complementary malaria control measures were under-utilized, depicted by over 90% of survey respondents denying having used these methods over the last one year. Ninety-three percent (93%) of the respondents had not sprayed their compounds using chemical insecticides while less than 9% reported having used repellents such as sprays, creams and mosquito coil.

#### Traditional methods

In the FGDs, it was reported that herbal medicines were used to treat various ailments. In some instances, herbal concoction was administered to entire family as a preventive measure against stomach infections and many parasitic diseases. For example, when an individual suffered from symptoms associated with malaria, vomiting and diarrhoea were induced using traditional medicines. Some people boiled roots, leaves or barks of some medicinal plants as a way of treating respiratory ailments and other infections as shown in the excerpt:

‘*Long time ago people used to slaughter a goat, make soup and mix with herbs then every member of the family would drink and they would be okay. Nowadays, it is a bit difficult, our children are very rigid when it comes to drinking traditional herbs…*’ Female participant, Salabani.

During FGDs, burning of plant leaves was reported to be effective in traditional houses “*Bororiet*”. However, only 8 % of respondents burnt cow dung or plant leaves to repel mosquitoes. This could be due to attitude changes against these traditional mechanisms as shown in the statement:

‘*We used to climb into the ‘Bororiet’, and then light a fire beneath so as to smoke out mosquitoes*. *When bed nets were brought, people became lazy to smoke leaves. In addition, we don’t want our clothes to smell smoke*.’ Female participant, Litein.

As reported during the FGDs, children and youth rejected traditional herbs formerly used to treat diseases such as malaria. Further, in two FGDs, some participants considered the aforementioned methods as backward as illustrated in the excerpt:

‘*People are educated and have become “digital”, you will not find them in the bushes uprooting plants and using them to smoke out mosquitoes*.’ Male participant Salawa.

### Barriers to effective malaria control in Baringo

#### Devolution of health services

The enactment of the Kenya Constitution (2010) marked the devolution of healthcare services from national to county governments. County governments became key actors in achieving objectives outlined in the revised Kenya Malaria Strategy 2009–2018. Established institutions such as the Division of Malaria Control (DOMC) was transformed into National Malaria Control Programme (NMCP) and Malaria Control Unit (MCU) at the national and county level respectively. This transition resulted in new challenges such as weak linkage between the two structures, low communication and inter-sector collaboration and inadequate funding of the county MCUs. According to informants, these challenges may undermine achievements made in malaria control as stated below:

‘*Although devolution is good, it seems it was done in a hurry. There were no proper structures in the counties. In addition, decision-making at the county level is done by the politicians rather that technocrats. In such system, you find more financial allocation given to infrastructure rather than service delivery.*’ Key informant, sub-county Malaria Control Unit.

‘*When we tell the county government to procure commodities for “malaria in pregnancy”, they say the partners are supposed to organize for that through the national government. It’s not clear how to go about it in the county and when you go to national government whom do you talk to yet health is devolved.*’ Key informant, sub-county Malaria Control Unit.

#### Financial constrains

It was reported (seven out of 12 key informants) that Baringo County government’s health allocation was lower than the county’s health requirements. This resulted in periodic shortage of malaria drugs and Rapid Diagnostic Test (RDT) kits. Inadequate supply of bed nets, shortage of antimalarial drugs and RDT kits were cited as key challenges for sustained malaria prevalence in the riverine zone as seen in the statement:

‘*Right now, I am conducting surveys on malaria status and most facilities do not have first line drugs for malaria and even for treating severe malaria cases.*’ Key informant, sub-county Malaria Control Unit.

All (12) key informants concurred that most health facilities had inadequate staff. For instance, it was reported that most dispensaries had only one nurse while health centres operated with about two nurses. In addition, some dispensaries were closed when a nurse in-charge was away on official duty or when he/she went for an annual leave as expressed:

‘*For us that is the norm… In fact, having one (nurse) is far much better. You would rather have that one even if she (nurse) will close for some time, during the months that she is present she assists*. *It has been a challenge though*…’ Key informant, Department of Health Services.

#### Limited research

Key informants revealed that malaria research was restricted to the lowland areas as compared to the riverine zone. This may have been due to accessibility and the fact that some parts of the lowland zone were considered as malaria hotspots as illustrated:

‘*The data we have is not convincing enough to allow other parts of Baringo County to get some supplies like the mass nets. This might call for another scientific study to help classify Baringo accordingly since the last study was done in 2004/2005*.’ Key informant, County Disease Surveillance.

### Mechanisms for communication and information exchange

Thirty four percent (34%) of the survey respondents mentioned family as their main source of malaria information (Table 3). Other noticeable sources of malarial information were radio and health facility, each at 16%. Most male respondents received malaria information from radio while most women relied on family members. Although female respondents depended on their family members for information, statistics confirmed that no gender was advantaged in terms of malaria information. (χ^2^ = 2.139, df = 1, *p* > 0.05).

**Table 3:** Sources of malaria information from survey respondents (*n* = 136)

	Respondents	
Source	n	(%)
Family	46	33.8
Other	7	5.1
Friends	12	8.8
Radio (national)	22	16.2
Television	6	4.4
Posters/pamphlets	6	4.4
School text books	2	1.5
Health facility	22	16.0
Community health worker/public health official	13	9.6
Total	136	100

This study found that 76% of survey respondents had not received any malaria teaching during the past one year. This finding is supported by in-depth interviews where nine out of 12 key informants asserted that community education and training were rare and conducted only during immunization campaigns, or when there was an imminent disease outbreak such as Cholera or Rift Valley Fever as expressed below:

‘*We haven’t had community education or trainings for quite some time now. So, if we do not get regular information on how to protect ourselves against malaria then it becomes a bit difficult. I can say there is a problem... If our people could get that (information), I know they would help pass the information to other community members*.’ Community resource person, lowland zone.

Key informants reported that public training and awareness campaigns were primarily conducted during local forums (*Baraza)*. In the riverine zone, structured *Baraza* were scheduled every fortnight while in the lowland zone they were ad hoc. Irrespective of the nature, *Baraza* was reported (in FGDs) as an effective platform for communication and information exchange among people living in Baringo County. According to key informants, community action days were additional avenues used to convey information on public health issues such as immunization campaigns. Community health volunteers together with officials from the department of health services spearheaded activities during community action days. In some areas, information was delivered by word of mouth by friends and opinion leaders.

### Complementary strategies needed

This study revealed that communities in Baringo County have limited options for malaria control and prevention. From 92% of survey respondents, and in all FGDs, it was apparent that the locals were willing to adopt complementary mechanisms for malaria control. These sentiments were expressed in the following quotes:

‘*Nets helps us only when sleeping and all other times before we retire to bed we are outside doing other businesses, so, if the government has other ways we are ready to receive*.’ Female participant, Salabani.

‘*More methods should be added because each day is different; today differs from tomorrow so we need more (options). If there is information then we need to be given, then we will know how the world is moving. We used to sleep up there (in the Bororiet), then we descended to the beds and we don’t know where we will be tomorrow…*’ Male participant, Kapkuikui.

### Framework for malaria control for communities in Baringo County

#### Participatory malaria risk assessment

Rainfall, rainy season, warm weather conditions and floods were key climate hazards linked to malaria upsurge in Baringo County. Erratic rains, rainy season, frequent floods especially in the lowland zone were hazard indicators of significant concern to the stakeholders. Biological hazards identified were high mosquito abundance and the presence of *Plasmodium falciparum.*

Community vulnerabilities were categorized into biological, socio-economic, institutional, physical and environmental. Vulnerability indicators such as large household size, number of people exposed to malaria (e.g. older children and other family members) were among the social indicators identified. The survey conducted found an average of four children per household where only one child had the privilege of sleeping under a bed net. This shows that more than half of family members were exposed to mosquito bite every night. Other indicators including high population density especially in the lowland, low-levels of education, the majority having primary education, high poverty index at 58.5%, low bed net ownership, large eave spaces, and irrigation activities were identified during in-depth interviews and FGDs. In this study, bed net ownership seemed to be influenced by a combination of accessibility and cost since 47% of those who owned bed nets got them from government facilities while 91% of those who bought the nets felt that they were not cheap. Other vulnerabilities reported during in-depth interviews were associated with community health seeking behaviour and non-compliance to malaria medication. For example, long distance travelled to hospitals (averagely 7 km) and long waiting hours at the health facility were the main reasons for delay by some people to seek health services. In addition, the low nurse-patient ratio was of concern to many stakeholders.

**Fig. 5:**
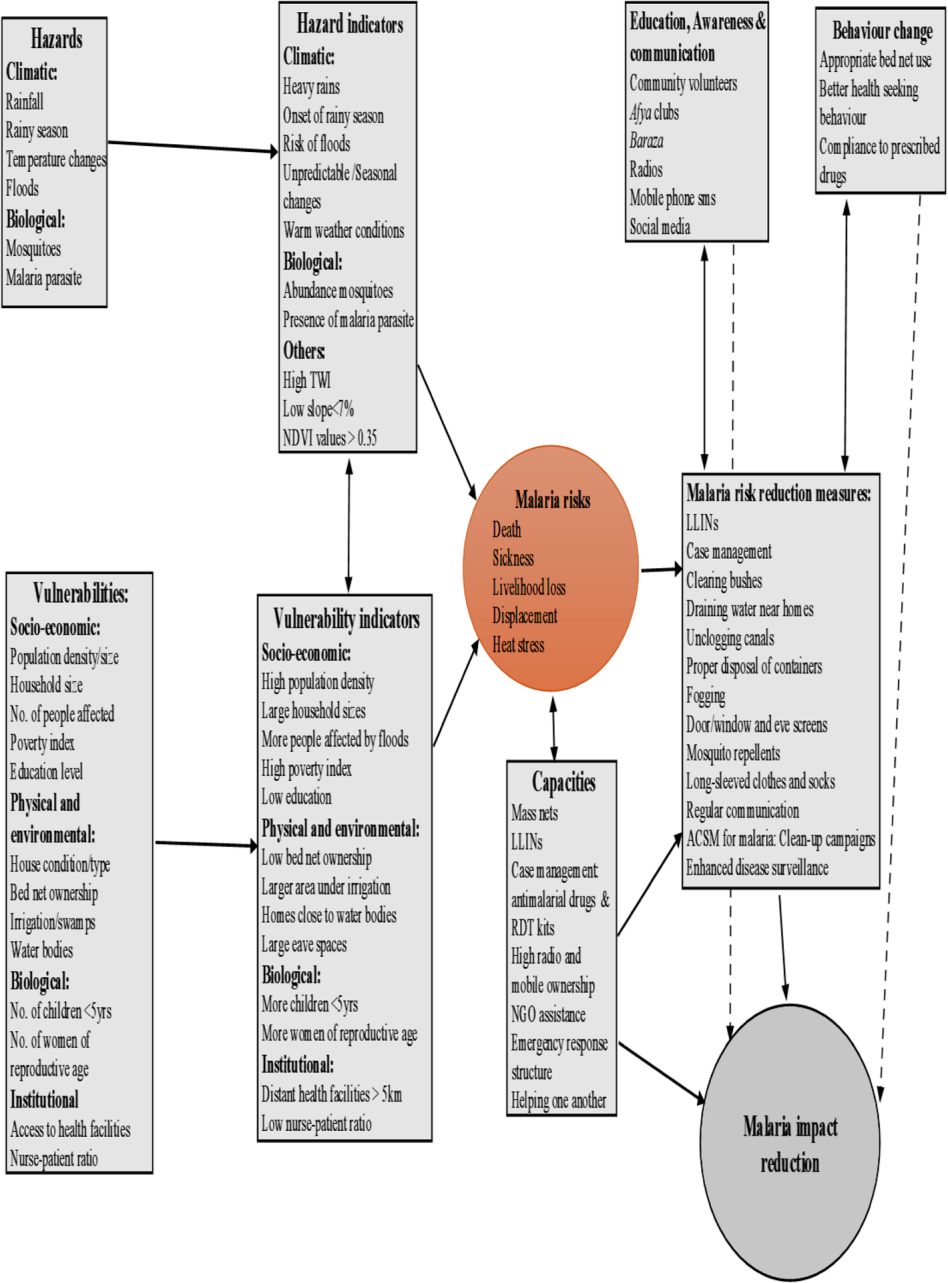
Malaria risk reduction framework developed by communities in Baringo County

Figure 5 illustrates malaria risk, vulnerability and capacity analysis process and malaria reduction measures suggested during participatory stakeholders’ forums. Risks identified were ranked as death, sickness, loss of working hours and livelihoods, displacement and heat stress. The risk of death was greatest among children and women living in distant or inaccessible villages. A participants mentioned that school going children spent a lot of time out of class either seeking medical service or due to ill-health. Another participant accounted that if an individual became sick it took about five days to recover. During this period, a sick person could not carry out their day-to-day activities.

#### Capacities to reduce malaria transmission in Baringo County

Participants cited malaria treatment service, vector control interventions such as LLINs, and communication technologies as capacities to deal with malaria in the region. Anti-malaria drugs and LLINs were key malaria control strategies. It was reported that when there was a shortage of anti-malaria drugs, the sub-county malaria coordinators redistributed medicines from low to high consumption facilities. This minimized chance of patients missing crucial medication. Availability of medicines for emergency response was an additional capacity among the medical practitioners to deal with imminent malaria outbreaks as illustrated below:

‘*Its (supply of antimalarial drugs) not adequate but we supplement by collecting from other facilities. If there is a shortage we request from other low volume facilities from the highlands areas.*’ Participant, Department of Health Services.

A participant suggested the use of radios and mobile phone short message service (SMS) arguing that these mechanisms were timely and effective for communication among community members. Despite the riverine zone being remote, the use of social media such as Twitter, WhatsApp and Facebook for communication was suggested. This is captured in the following statement:

“*But now, we are in a digital world, we do Twitter, WhatsApp and Facebook … Only a few people are not conversant with the social media.*” Community resource person, riverine zone.

The government’s mass nets and non-governmental organizations (NGOs) support were capacities available to communities in the lowland zone. Further, a participant reported that women support groups were common in the lowland zone where members assisted each other in the farms and other entrepreneurial activities. Participants concurred that such support groups could be strengthened to act as community organizations during implementation of malaria reduction strategies.

#### Malaria risk reduction measures

Proposed strategies for malaria control were LLINs and case management supplemented with community-based vector control measures comprising environmental management mechanisms as shown in Fig. 5. These measures included clearing bushes close to homes, opening blocked canals, recycling and proper disposal of containers. Other strategies comprised the use of repellents such as mosquito coil, cream, and indoor aerosols that the locals were willing to use. Participants agreed that while outdoors, wearing long sleeved clothes, long pants and socks were simple, yet effective ways that minimized mosquito bites. Participants concurred that locally available materials such as wall decorations could be used as screens for doors, window, and eave spaces. The use of screens was not new to the people living in the lowland zone since some community members had earlier benefited from screens donated by a research team working on Leishmaniasis.

The MCU through the sub-county malaria coordinators suggested that they could conduct strategic fogging in the lowlands where *Prosopis* bushes sustained high mosquito abundance. Local leaders concurred that they would encourage the people to continue with their cultural practices such as burning cow dung and plant leaves to kill or repel mosquitoes. They were informed that these remedies were locally available, less costly and effective in repelling biting insects. Local leaders from the lowland zone were encouraged to rid their dwellings of *Prosopis* as a measure to reduce contact with mosquitoes. Participants were informed and encouraged to take actions that would ensure they had good health rather than wait for the government to initiate such actions. It was agreed that the local administration would encourage the people to reduce the bushes around their dwellings by organizing periodic community *Prosopis* clearing days as a way of keeping the vector at bay.

A consensus was reached on using Advocacy Communication and Social Mobilization (ACSM) for malaria control. This would be achieved through *Baraza*, radios and mobile phone SMS that were at the disposal of most community members. Vernacular radio stations could be exploited to reach the rural communities with malaria messages. Participants were eager about using social media such as WhatsApp or Facebook for community communication arguing that these platforms provided explicit, graphical information that were easily understood.

The MCU together with county disease surveillance and public health departments committed to strengthening collaboration with other institutions for efficient health service provision in the county. The MCU officers affirmed that in collaboration with NMCP and other partners they would minimize delays in procurement and service delivery. Consultative deliberations with the county health executive led to the resolution that community health volunteers were to be recruited and trained in order to enhance community education and awareness in the inaccessible areas in the county. Once trained, these volunteers would act as community malaria ambassadors to drive behaviour change at household and community levels. School teachers were urged to initiate “*Afya*” (health) clubs in order to increase awareness amongst school children. Once equipped with information, the said groups would clarify existing misconceptions among families and community members.

## Discussion

This study shows significant increases in malaria cases during March, April, May, June and October in the riverine zone. In the lowland zone, non-significant decrease in malaria trends could be attributed to interventions especially the mass net distributions. Locals had enormous knowledge on the cause of malaria though there were misconceptions about the mode of malaria transmission. Misconceptions about malaria etiology were not unique to this study since similar perceptions have been reported among communities in western Kenya [28]. These misguided perceptions indicate a knowledge gap that potentially influence choices of malaria prevention or treatment pattern [29]. Thus, there is need for enhanced community knowledge though public education and awareness in order to increase uptake of preventive measures. Improved public awareness resulted in substantial reduction in malaria cases among rural communities in Malaysia [30].

This study identified several factors predisposing the locals to malaria risk. Most of these activities were gender-oriented and resulted in longer outdoors stays especially after dusk. These activities increased chances of contact with mosquitoes since *Anopheles* peak biting hours [31, 32] coincided with the duration when most people were outdoors. Outdoor occupation was reported to increase malaria transmission in Tanzania [33]. This study showed that very few people used personal protection measures while outdoors. When indoors, bed net was the dominant malaria control intervention. Nevertheless, prolonged outdoor stays may have decreased the effectiveness of bed nets since most people were likely to be bitten by mosquito before sleeping under the mosquito nets.

Consistent with the current study, burning of cow dung or local plant leaves were reported in Tanzania [33] and West Africa [34] as traditional mechanisms for repelling mosquitoes and other biting insects. A majority of community members had not used any mosquito repellents, or chemical sprays implying suboptimal utilization of complementary interventions for malaria prevention among the people in Baringo County. This could be attributed to over-reliance on mosquito nets and cultural transformation since those who used traditional mechanisms were considered retrogressive.

From this study, challenges stemming from devolved healthcare system were found to hinder effective malaria control in Baringo County. This was consistent with the gaps identified in the Malaria Communication Strategy 2016–2021 that further elucidated directions and approaches to address these issues [26]. The county MCU should play a lead role by coordinating the establishment of local structures requisite for the implementation of malaria reduction strategies. Further, the MCU should lobby for financial and technical support from the NMCP and partners in malaria control.

Operational communication and information exchange platforms are essential in enhancing community health education and awareness. This study revealed that majority of community members had not received malaria information over the past one year suggesting low-levels of community engagement on public health issues. Similar to these findings, it was reported that most rural communities depended on kin and radio for malaria information in Cameroon [35]. *Baraza* was identified as an effective platform for communication and information exchange among communities in Baringo. *Baraza* was lauded as efficient and community friendly platform for communication since the messages were in vernacular and potentially reached a large number of people. If strengthened and properly utilized, *Baraza* could help achieve significant community awareness not only for malaria control but public health awareness and education.

Despite having numerous strategies for malaria control and prevention in Kenya [9], this study showed that these strategies were underutilized in Baringo County. Taking into consideration the challenges associated with LLINs and case management strategies, community-driven supplementary approaches need to be introduced in order to minimize malaria burden among the rural communities and achieve a malaria-free Kenya. Concerns over inadequate bed nets as well as possible outdoor malaria transmissions fuelled the quest to adopt additional methods for malaria control. Evidently, this called for diversification and adoption of complementary interventions.

This study presents for the first time a framework of malaria control for communities living in Baringo, Kenya. If implemented, the measures identified would help meet strategic objectives outlined in KMS 2009–2018. Local structures for implementation included ACSM platforms and local organizations. Enhanced utilization of local malaria preventive measures can significantly contribute in closing the gaps associated with primary intervention strategies.

Several complementary mechanisms were identified and proposed for adoption in order to reduce malaria risk in Baringo. Besides the LLINs and case management mechanisms, complementary strategies such as social mobilization for environmental management were recommended. These activities included bush clearing, unclogging drainage canals and proper disposal of empty containers. Outdoor protection against mosquito bites using long sleeved clothes, long pants and socks were cited as simple strategies that could effectively minimized outdoor malaria transmission. A study found that insecticide-treated clothing were effective in controlling the vector of Dengu and Zika virus [36]. Using locally accessible materials as screen for doors, windows and eave spaces would minimize indoor mosquito densities. Window and door screens were effective in controlling *indoor resting mosquitoes in Tanzania* [37]*. Similar mechanisms were suggested for control of malaria transmission in India* [38]*.*

The ACSM mechanism is a key component of any integrated vector control approach. The ACSM for malaria control is an indispensable tool for a community-based approach [17]. In this framework, *Baraza*, radios and mobile phone SMS were identified as ACSM platforms for malaria communication. In addition, the fast-growing social media such as Facebook, Twitter or WhatsApp could be exploited to pass malaria messages and information to individuals and groups. The ACSM mechanisms such as radio, television and cell phones have been used for social mobilization campaigns for tuberculosis [39] and malaria control [40]. The Kenya Malaria Communication Strategy 2016–2021 recommended that these social media spaces be taken up by county governments as potential social mobilization and interpersonal communication mechanisms for malaria communication [26].

Community health volunteers and school health “*Afya*” clubs could spearhead social mobilization for behaviour change consequently demystifying existing myths and misconceptions. Studies have shown that the use of school children as health messengers led to improved knowledge and practices among communities in India [41]. A malaria-free Kenya will require implementation of community-driven integrated approaches for malaria reduction. The framework may be applied selectively in other settings. The integrated approaches have been applied at the Kenyan coast [42] and in other parts of the world for malaria control [43]. Community-driven malaria prevention and control interventions would minimize outdoor malaria transmission, and ameliorate the challenges of LLINs and case management currently experienced in Baringo County. Collectively, these interventions would result in substantial reduction in malaria burden.

## Conclusions

This study analysed malaria trends, assessed lay knowledge and developed a community-based malaria control framework for communities in Baringo County, Kenya. Malaria trends increased during 2005–2014 in the riverine zone while there was no change in malaria cases reported in the lowland zone. The knowledge gap on malaria etiology should be addressed to minimize impact and enhance uptake of appropriate malaria management mechanisms. Successful malaria control requires community-driven implementation of diverse strategies as well as robust institutional support that reduce malaria prevalence by minimizing both indoor and outdoor malaria transmissions.

## Abbreviations

ACSM: Advocacy Communication and Social Mobilization
ACTs: artemisinin-based combination therapies
CMDRM: Community Based Disaster Risk Management
DOMC: Division of Malaria Control
FGDs: focus group discussions
IPTp: Intermittent Preventive Treatment in pregnancy
IRS: Indoor Residual Spraying
ITNs: Insecticide-Treated Bed Nets
IVM: Integrated vector management
KIIs: key informant interviews
KMS: Kenya Malaria Strategy
LLINs: Long Lasting Insecticidal Nets
MCS: Malaria Communication Strategy
MCU: Malaria Control Unit
NACOSTI: National Commission for Science, Technology, and Innovation
NGOs: non-governmental organizations
NMCP: National Malaria Control Programme
RDT: Rapid Diagnostic Test
SMS: short message service
WHO: World Health Organization
